# The two shapes of the Tau protein

**DOI:** 10.7554/eLife.38516

**Published:** 2018-07-10

**Authors:** Jeffery W Kelly

**Affiliations:** 1 Department of ChemistryThe Scripps Research InstituteLa JollaUnited States; 2 Department of Molecular MedicineThe Scripps Research InstituteLa JollaUnited States

**Keywords:** tau, Alzheimer's disease, protein aggregates, seeding, amyloid fibers, transthyretin, Human

## Abstract

Tau proteins can convert from an inert shape to a misfolded shape that seeds the growth of fibers that contribute to the pathology of Alzheimer’s disease.

**Related research article** Mirbaha H, Chen D, Morozova OA, Ruff KM, Sharma A, Liu X, Goodarzi M, Pappu RV, Colby DW, Mirzaei H, Joachimiak LA, Diamond MI. 2018. Inert and seed-competent tau monomers suggest structural origins of aggregation. *eLife* **7**:e36584. doi: 10.7554/eLife.36584

Most of the time, proteins fold into a single stable shape to perform their role in the body, but occasionally they can adopt a different conformation. These 'misfolded' proteins can be associated with a range of degenerative conditions known as amyloid disorders, which includes the transthyretin amyloidoses as well as Alzheimer’s and Parkinson's diseases. This is because the misfolded proteins go on to stick together and form toxic insoluble aggregates, for example ‘amyloid’ fibers, that accumulate inside cells. One such protein is Tau, which aggregates in people with Alzheimer’s disease. It is thought that the misfolded Tau proteins and the various Tau aggregates, including amyloid fibers, contribute to the onset of Alzheimer’s disease (Eisele et al., 2015), but these processes are not fully understood.

Inside a cell, the harmful aggregation process is believed to begin with a ‘seed’, a template that can trigger the assembly of a given protein. These seeds are thought to be crucial in the spread of the disease. The hypothesis is that seeds can convert the normally folded protein into an aggregate of the same protein, before cells release them into the environment for neighboring cells to take up (Eisenberg and Jucker, 2012). This could be how diseases linked to the Tau protein, such as Alzheimer’s, propagate from one cell to another; there, the aggregates would travel through the brain using the connections between neurons (Clavaguera et al., 2009; Sanders et al., 2014). While the identity of the seeds remains unclear, until now, almost all scientists have believed that they are an assembly of a given individual misfolded protein. Now, in eLife, Marc Diamond of the University of Texas Southwestern Medical Center (UTSW) and colleagues – including Hilda Mirbaha as first author – report the existence of a stable of form of an individual Tau protein that can start the aggregation process on its own (Mirbaha et al., 2018).

The fact that the seed may not be an assembly of a misfolded protein, but instead be a single protein – a monomer – with a different conformation had only been suggested twice before. In 2005, a study proposed that a change in the conformation of a Tau monomer had a critical role in triggering the process of aggregation (Chirita et al., 2005). And in 2011, it was hypothesized that the aggregation of the huntingtin protein, which is involved in another amyloid disorder known as Huntington’s disease, could start with a single protein (Kar et al., 2011). However, in these two studies the monomer that could initiate the seeding process was not isolated and studied. Despite robust data interpretation, many in the scientific community dismissed the idea of monomeric seeds, reluctant to challenge the widely ingrained concept that they are instead an assembly of a misfolded protein.

So far, Tau was considered to be an intrinsically disordered protein – more like a flexible noodle than a protein with a well-defined and stable, three-dimensional structure (Schweers et al., 1994). Instead, Mirbaha et al. show that the Tau protein can fold into two distinct and fairly well-defined conformations. One of these shapes is stable, nontoxic and does not easily aggregate; the other acts as a seed and can help to convert another ‘harmless’ Tau monomer into a misfolded Tau that will form toxic aggregates by seeding or self-assembly. In addition, Tau can very slowly change from the inert to the seed-competent conformation. It is known that small molecules can bind to the inert conformation of proteins that are prone to misfolding, and thus prevent the conformational change that leads to amyloid diseases (Johnson et al., 2012).

For example, transthyretin is another protein with two ways of folding, and whose toxic conformation damages various nervous systems, as well as the heart. However, drugs known as kinetic stabilizers can slow down the degenerative process by increasing the population of the properly folded conformation. More precisely, three placebo-controlled clinical trials showed that small molecules, such as the drugs tafamidis and diflunisal, can bind to the non-pathogenic form of transthyretin and stabilize it, which prevents the protein from converting into the conformation that initiates aggregates and leads to degenerative pathologies (Coelho et al., 2012; Berk et al., 2013; Rosenblum et al., 2018). This suggests that it should be possible to fashion similar kinetic stabilizers for the Tau protein, and offer better treatment for diseases such as Alzheimer’s.
